# Prevalence of *Mycoplasma genitalium* in men with urethritis in a large public hospital in Brussels, Belgium: An observational, cross-sectional study

**DOI:** 10.1371/journal.pone.0196217

**Published:** 2018-04-26

**Authors:** Agnès Libois, Marie Hallin, Tania Crucitti, Marc Delforge, Stéphane De Wit

**Affiliations:** 1 Department of Infectious Diseases, CHU Saint-Pierre, Université Libre de Bruxelles (ULB), Brussels, Belgium; 2 Center for Molecular Diagnostics, Laboratoire Hospitalier Universitaire de Bruxelles, Brussels, Belgium; 3 Department of Clinical Sciences, Institute of Tropical Medicine (ITM), Antwerp, Belgium; GGD Amsterdam, NETHERLANDS

## Abstract

**Background:**

*Mycoplasma genitalium* (MG) is a cause of urethritis. While resistance to azithromycin is increasing, routine detection of MG is not performed in Belgium, where its prevalence is unknown. The aim of this study is to determine prevalence of MG in men with urethritis.

**Method and findings:**

An “in-house” amplification assay detecting MG was performed on urine of men with complaints of urethritis who consulted the emergency unit or the Sexually Transmitted Infection clinic of our public hospital in Brussels. *Chlamydia trachomatis* (CT) and *Neisseria gonorrhoeae* (NG) were tested on the same sample. A total of 187 men were tested. Prevalence of MG was 9% (95% Confidence Interval: 5 to 13.2%). CT was detected in 20%, NG in 22% and 56% of samples were negative for these three pathogens. Neither age, ethnic origin, sexual orientation nor HIV infection were associated with MG urethritis.

**Conclusion:**

*M*. *genitalium* was identified in 9% of men with complaints of urethritis indicating that amplification assay detecting MG should be implemented in routine testing for those patients.

## Introduction

*Mycoplasma genitalium* (MG) is a cause of urethritis in men and is associated in women with cervicitis, pelvic inflammatory disease (PID) and infertility. [1]

Prevalence of MG in the general population is low, ranging from 0.8% in clinic based samples from asymptomatic patients to 3.2% in MSM in the community; while in men with nonchlamydial-nongonococcal urethritis (NCNGU), it varies between studies from 15% to 35%. [2, 3]

In Europe, in men with urethritis, prevalence varies between 6% and 16.7%. [4, 5]

Due to the increasing resistance of MG to azithromycin (40% in London and Denmark, 14% in France) [2,4,6], azithromycin 1 g is replaced by 1 week of doxycycline as first-line therapy for nongonococcal urethritis (NGU) in the 2016 European guideline. [7] Doxycycline, however, has poor efficacy on MG with cure rates of 31% to 45%. [1] Moxifloxacin is recommended after a treatment failure with azithromycin but resistance is also increasing (reaching 13% in Australia). [1,4,8]

Nucleic acid amplification is the only clinically useful method to detect MG. No FDA-approved test is available yet. Therefore, detection of MG is not recommended by US guidelines and still not routinely performed. [9] However, the European guideline recommends these tests in men with urethritis, when available. [7,10] New tests including detection of azithromycin resistance in MG were recently CE labeled. [11,12]

The high prevalence of MG among NGU and its capacity to develop resistance are likely to worsen in the future, jeopardizing empirical treatment options for NGU. In Belgium, the prevalence of MG is unknown. Our aim was to determine its prevalence in men with complaints of urethritis in a large public urban hospital in Brussels.

## Methods

### Patients

This was an observational, cross-sectional study targeting men with complaints of urethritis (dysuria, urethral discomfort and/or urethral discharge) presenting at the emergency unit or at the Sexually Transmitted Infection clinic of University Saint-Pierre hospital in Brussels, between 1/10/2012 and 11/02/2014.

Patients reporting any antibiotic treatment in the previous 30 days were excluded.

#### Pathogen detection and urine leucocytes count

First-void urines were collected and split into two aliquots: the first, inoculated in a sterile tube without conservative, was used for urinary sediment analysis and dipstick test; the second was inoculated into a multi-Collect transport medium tube (Abbott, Maidenhead, UK) for *C*. *trachomatis* (CT), *N*. *gonorrhoeae* (NG) and MG detection.

Urine sediment analysis was performed using the flow cytometer Sysmex UF-100. Tubes were then used for a dipstick test on the Aution Max AX-4280 (Menarini). CT and NG were tested using the Abbott RealTime CT/NG assay on the Abbott *m*2000 automated platform. Samples were then stored at -20°C. The amplification assay detecting MG was performed by series: DNA was extracted using the Abbott platform according to the manufacturer’s instructions. A validated in-house real-time PCR amplifying a fragment of the *pdh*D gene was then performed. The in house real-time PCR for detection of *M*. *genitalium* was performed based on the assay described by Müller et al. [13] and according to the procedure summarized elsewhere [14].

As MG identification was performed by testing series of samples, physicians did not receive the result, treatment of patients was thus not influenced by the presence or not of *M*. *genitalium* infection in the urine test.

### Definition

Objective urethritis was defined as a discharge on exam and/or urine leucocytes ≥35cells/μl and/or a positive leucocyte esterase. A leucocytes count ≥35cells/μl corresponds to 10 leucocytes per high power field using microscopic examination of urine after centrifugation, the recommended limit value to document urethritis according to US guidelines. [9,15]

### Statistical analyses

Simple descriptive statistics were used for summarized characteristics.

For comparison, Mann-Whitney U test was used for continuous data and Fisher’s exact test for categorical data. All reported p-values are two-sided. Analyses were produced using the SAS statistical software (version 9.4; SAS Institute, Cary, NC, USA).

### Ethics

The study was approved by the local Ethics Committee (OM007, study B076201214623) and the Institutional Review Board of Institute of Tropical Medicine. An oral inform consent was given by all participants.

## Results

During a 16 months period, 187 men with complaints of urethritis were included. Patients characteristics and results of urine tests are shown in Table 1.

**Table 1:** Demographics, clinical features and results of urine samples among 187 patients with complaints of urethritis.

	Patients with complaints of urethritis N = 187	Patients with objective urethritis N = 93	Patients with complaints but no objective urethritis N = 43	Patients with complaints but incomplete data N = 51
Age (years), median (interquartile range)	30 (26–38)	29 (23–37)	31 (26–39)	31 (27–39)
Caucasians	81%	73%	93%	88%
Heterosexual	64%	65%	64%	64%
Men who have sex with men	36%	35%	36%	36%
HIV positive	7%	4%	12%	8%
Urine PCR-NG positive. n(%)	42 (22%)	38 (41%)	1 (2%)	3 (6%)
Urine PCR-CT positive. n (%)	37 (20%)	29 (31%)	1 (2%)	7 (14%)
Urine PCR-*M*.*genitalium* positive. n (%)	17 (9%)	10 (11%)	1 (2%)	6 (12%)
No pathogen found. n (%)	104 (56%)	29 (31%)	40 (93%)	35 (69%)

Prevalence of *M*. *genitalium* was 9% (95% Confidence Interval (CI): 5 to 13.2%) on the whole cohort, 11% in NGU (95% CI: 4.5 to 17) and 13% in NCNGU (95% CI: 2.9 to 20.6), excluding coinfection (MG and infection with CT and/or NG). Nine men had a NG-CT co-infection, two had a CT-MG co-infection and one a NG-CT-MG co-infection. No pathogen was found in 56% of samples. Men ≥30 years were more at risk of negative results for the 3 bacteria tested, whatever their sexual orientation (65% vs 45%, p = 0.009)

Diagnosis of *M*. *genitalium* or *N*.*gonorrhoeae* infection were not significantly associated with age, ethnicity, sexual orientation or HIV infection.

CT was more frequently identified in men <30 years (27% versus 14% in men >30 years, p = 0.0415) and in heterosexual men (27% vs 8% in MSM, p = 0.0066, also statistically significant in multivariate analysis).

Half of the men (93/187) had an objective urethritis: urine leucocytes ≥35/μl in 54 of 104 urine sediment performed, six patients had leucocytes <35/μl but a positive leucocyte esterase and 33 had a discharge on exam and no urine sediment analysis performed.

Among patients with objective urethritis, MG was present in 11%, 16% of NGU and 27% of NCNGU. The three patients with a coinfection with MG (two with CT and one with CT and NG) had an objective urethritis. No pathogen was found in 31% of samples. This percentage was also higher in men ≥30 years in this subgroup of patients with objective urethritis (42% vs 21%, p = 0.026).

Among the 43 men with complaints but no objective urethritis, only 3 had a bacteria detected (1 MG, 1 CT, 1 NG).

Finally, 51 men presented complaints but no objective discharge and due to missing data (urine leucocytes or leucocytes esterase were not performed), they could not be classified as having or not having an objective urethritis.

## Discussion

In our population of men with complaints of urethritis, prevalence of MG was 9% on the whole cohort and 11% in patients with objective urethritis. To our knowledge, these are the first Belgian data on MG in men.

These prevalence rates are similar to those found in men with objective NGU from recent series: 16.7% in London [4] and 11.9% in Tel Aviv [16]. In Amsterdam, prevalence in men reporting symptoms of urethritis was 5.9%. [5] The high prevalence we found and the increase of azithromycin resistance in numerous studies confirms the need for routine detection of MG in patients with urethritis, preferably combined with resistance testing.

Introducing these tests in daily practice in Belgium is difficult as they are not reimbursed by the social security and there is no FDA approved, standardized or well validated commercially available test (CE labeled kits suffer from limited validation). [10]

A limitation of our study is that a substantial number of patients did not carry out all tests necessary to objectify urethritis.

No control group was included in our study but prevalence is known to be low in the general population including in clinical based samples from asymptomatic patients. [3]

This was confirmed in our setting: 77 men (with or without complaints of urethritis) were tested in our STI clinic and none was positive for *M*.*genitalium* among asymptomatic men. (Mahadeb B, unpublished data).

No resistance testing for azithromycin was performed in our study as we had no access to an assay identifying the point mutations associated with azithromycin resistance at the time of the study. A very recent Belgian study in female sex workers showed a prevalence of MG of 10.8% and macrolide-resistance associated mutations (A2058G and A2059G) in 6.5% of isolates. [17]

Further studies in Belgium should address the prevalence of macrolide and moxifloxacin resistance in men with urethritis.

In conclusion, MG was identified in 9% of men with complaints of urethritis and in 11% of men with objective urethritis. Amplification assays detecting *M*. *genitalium* should be implemented in routine testing for men with urethritis, preferably with resistance testing, allowing a more adapted antibiotic treatment.

## Supporting information

S1 File (XLS)
